# Importance of Taiman in Larval-Pupal Transition in *Leptinotarsa decemlineata*

**DOI:** 10.3389/fphys.2019.00724

**Published:** 2019-06-13

**Authors:** Qing-Yu Xu, Jun-Li Du, Li-Li Mu, Wen-Chao Guo, Guo-Qing Li

**Affiliations:** ^1^Key Laboratory of Integrated Management of Crop Diseases and Pests, Ministry of Education, College of Plant Protection, Nanjing Agricultural University, Nanjing, China; ^2^College of Agriculture, Anhui Science and Technology University, Fengyang, China; ^3^Institute of Plant Protection, Xinjiang Academy of Agricultural Sciences, Urumqi, China; ^4^Key Laboratory of Intergraded Management of Harmful Crop Vermin of China North-Western Oasis, Ministry of Agriculture, Urumqi, China

**Keywords:** *Leptinotarsa decemlineata*, *LdTai*, metamorphosis, trachea, fat body

## Abstract

Insect Taiman (Tai) binds to methoprene-tolerant to form a heterodimeric complex, mediating juvenile hormone (JH) signaling to regulate larval development and to prevent premature metamorphosis. Tai also acts as a steroid receptor coactivator of 20-hydroxyecdysone (20E) receptor heterodimer, ecdysone receptor (EcR) and Ultraspiracle (USP), to control the differentiation of early germline cells and the migration of specific follicle cells and border cells in ovaries in several insect species. In holometabolous insects, however, whether Tai functions as the coactivator of EcR/USP to transduce 20E message during larval-pupal transition is unknown. In the present paper, we found that the *LdTai* mRNA levels were positively correlated with circulating JH and 20E titers in *Leptinotarsa decemlineata*; and ingestion of either JH or 20E stimulated the transcription of *LdTai*. Moreover, RNA interference (RNAi)-aided knockdown of *LdTai* at the fourth (final) instar stage repressed both JH and 20E signals, inhibited larval growth and shortened larval developing period. The knockdown caused 100% larval lethality due to failure of larval-pupal ecdysis. Under the apolysed larval cuticle, the *LdTai* RNAi prepupae possessed pupal thorax. In contrast, the process of tracheal ecdysis was uncompleted. Neither JH nor 20E rescued the aforementioned defectives in *LdTai* RNAi larvae. It appears that Tai mediates both JH and 20E signaling. Our results uncover a link between JH and 20E pathways during metamorphosis in *L. decemlineata*.

## Introduction

Insect development and reproduction are essentially governed by the steroid 20-hydroxyecdysone (20E) and the sesquiterpenoid juvenile hormone (JH) (Jindra et al., 2013, 2015a; Santos et al., 2019). During larval development in holometabolous insects, the presence of JH suppresses the expression of metamorphosis-initiation genes and 20E can only induce the larval-larval molt (Riddiford, 1994; Ureña et al., 2014). At the late stage of the final instar larva, JH titer drops substantially, to terminate the suppression effects on metamorphosis-initiation genes (Smykal et al., 2014b). As a result, 20E induces larval-pupal and pupal-adult molts (Jindra et al., 2013; Santos et al., 2019).

JH acts through its receptor methoprene-tolerant (Met) (Ashok et al., 1998; Charles et al., 2011; Jindra et al., 2015b), a basic helix-loop-helix (bHLH)/Per-Arnt-Sim (PAS) transcription factor (Partch and Gardner, 2010). After binding of JH, Met recruits another bHLH/PAS protein Taiman (Tai) to form an active receptor complex. The binding of Met/Tai heterodimer to JH response element (JHRE) triggers the transcription of *Krüppel-homolog 1* (*Kr-h1*) and *Hairy* to repress metamorphosis (Saha et al., 2016; Wu et al., 2016), partially by inhibition of prothoracicotropic hormone (PTTH) signaling (PTTH-Torso-MAPK) to repress 20E biosynthesis and release in Coleopteran for instance the Colorado potato beetle *Leptinotarsa decemlineata*, the most destructive insect defoliator of potatoes (Meng et al., 2018, 2019), and Lepidopteran such as the tobacco hornworm *Manduca sexta* (Rountree and Bollenbacher, 1986; Watson and Bollenbacher, 1988).

In insects, Tai also acts as a steroid receptor coactivator of the functional 20E receptor heterodimer, ecdysone receptor (EcR) and Ultraspiracle (USP) in adults (Yao et al., 1993; Bai et al., 2000). In the common fruit fly *Drosophila melanogaster*, 20E-dependent binding of Tai to EcR/USP is crucial for the migration of specific follicle cells and border cells in ovaries, and for the differentiation of early germline cells (Bai et al., 2000; König et al., 2011; König and Shcherbata, 2015). In the yellow fever mosquito *Aedes aegypti* (Liu et al., 2018) and the firebug *Pyrrhocoris apterus* (Smykal et al., 2014a), depletion of *Tai* impairs oocyte development.

Is Tai involved in the development of juvenile insects, as a coactivator of EcR/USP? In the German cockroach *Blattella germanica* (Lozano and Belles, 2014) and *P. apterus* (Smykal et al., 2014b), knockdown of *Met* in nymphal instars triggers precocious metamorphosis, whereas depletion of *Tai* causes nymphal lethality (Lozano et al., 2014; Smykal et al., 2014a). The different negative effects between Met- and Tai-depleted insects suggest that Tai may be an important component in 20E transduction cascade regulating insect development in hemimetabolan insects.

In holometabolan insects, whether Tai functions as a coactivator of EcR/USP during larval-pupal-adult transition is unknown. In the oriental fruit fly *Bactrocera dorsalis*, depletion of *Tai* in larval stage produces no mortality but causes precocious larvae-pupae development (Liu et al., 2017), a typical phenotype in JH deficient insects (Jindra et al., 2013, 2015a). In the red flour beetle *Tribolium castaneum* (Bitra et al., 2009) and *D. melanogaster* (Perrimon et al., 1996; Liu et al., 2009; Abdou et al., 2011; König et al., 2011; Jindra et al., 2015b; Wang et al., 2016), RNAi of or mutation in either *Met* or *Tai* causes similar larval lethality. Since JH regulates almost every aspect of an insect’s life, it is likely that depletion of either Met or Tai in *T. castaneum* and *D. melanogaster* attenuates JH signaling, and results in severe larval lethality. Therefore, the specific role of Tai as a coactivator of EcR/USP during larval-pupal-adult transition remains unproven in any holometabolous insects.

In *L. decemlineata*, the main genes that are involved in ecdysteroidogenesis (Wan et al., 2013; Kong et al., 2014), JH biosynthesis (Fu et al., 2016; Li et al., 2016), 20E (Liu et al., 2014; Guo et al., 2015, 2016; Xu et al., 2018a,b, 2019a,c) and JH (Meng et al., 2018, 2019) signaling have been identified. Knockdown of *LdMet* reduces the body size and accelerates larval development (Meng et al., 2018, 2019), whereas 20E deficient (Kong et al., 2014) or repression of 20E signaling (knockdown of *LdEcR, LdUSP, LdHR3, LdHR4, LdE74, LdE75*, or *LdFTZ-F1*) (Liu et al., 2014; Guo et al., 2015; Shi et al., 2016; Xu et al., 2018a,b, 2019a,c) causes failure of larval-pupal-adult ecdysis and lethality. The distinct phenotypes between disturbance of JH and 20E signals will facilitate us to determine the roles of Tai during larval-pupal development. In the present paper, we first measured the transcription patterns of *LdTai* at whole development excursion. We then examined the defects after silence of *LdTai* using RNAi, and found that the silence repressed both JH and 20E signals and brought about lethality due to failure of larval-pupal ecdysis. Moreover, the process of tracheal ecdysis was uncompleted in *L. decemlineata*. Our results unveil, for the first time in a holometabolous insect species, that Tai mediates both JH and 20E signaling pathways during larval-pupal transition.

## Materials and Methods

### Insect Rearing

The *L. decemlineata* beetles were kept in an insectary according to a previously described method (Shi et al., 2013), with potato foliage at the vegetative growth or young tuber stages in order to assure sufficient nutrition. At this feeding protocol, the larvae progressed through four distinct instars, with approximate periods of the first-, second-, third-, and fourth-instar stages of 2, 2, 2 and 4 days, respectively. Upon reaching full size, the fourth larval instars stopped feeding, dropped to the ground, burrowed to the soil and entered the prepupal stage. The prepupae spent an approximately 3 days to pupate. The pupae lasted about 5 days and the adults emerged.

### Preparation of dsRNAs

Specific primers used to clone the fragments of dsRNAs derived from *LdTai* and *enhanced green fluorescent protein* (*egfp*) were listed in Supplementary Table S1. These dsRNAs were individually expressed using *Escherichia coli* HT115 (DE3) competent cells lacking RNase III following an established method (Kong et al., 2014). Individual colonies were inoculated, and grown until cultures reached an OD600 value of 1.0. The colonies were then induced to express dsRNA by addition of isopropyl β-D-1-thiogalactopyranoside to a final concentration of 0.1 mM. The expressed dsRNA was extracted and confirmed by electrophoresis on 1% agarose gel. Bacteria cells were centrifuged at 5000 × *g* for 10 min, and resuspended in an equal original culture volume of 0.05 M phosphate buffered saline (PBS, pH 7.4). The bacterial solutions (at a dsRNA concentration of about 0.5 μg/ml) were used for experiment.

### Bioassay

20E (Sigma-Aldrich, United States) or JH (Sigma-Aldrich, United States) was dissolved in distilled water with added surfactant (Tween 20, 1 g/L) to give stock solutions of 100 ng/ml. It was diluted ten folds with distilled water before bioassay.

Two independent bioassays were carried out as previously described (Kong et al., 2014) using newly ecdysed fourth-instar larvae. For each bioassay, seven treatments were set: PBS, ds*egfp*, ds*egfp*+10 ng/ml 20E (JH), ds*Tai*-1, ds*Tai*-1+10 ng/ml 20E (JH), ds*Tai*-2, and ds*Tai*-2+10 ng/ml 20E (JH). Potato leaves were immersed with a bacterial suspension containing a dsRNA, or dsRNA+20E (JH) for 5 s, removed, and dried for 2 h under airflow on filter paper. Five treated leaves were then placed in Petri dishes (9 cm diameter and 1.5 cm height). The newly ecdysed fourth-instar larvae were starved for at least 4 h prior to the experiment. Then, ten larvae were transferred to each dish as a repeat. The larvae were firstly allowed to ingest potato foliage immersed with PBS, ds*egfp*, ds*Tai*-1, or ds*Tai*-2 for 2 days, and then consumed leaves dipped with PBS, ds*egfp*, ds*egfp*+10 ng/ml 20E (JH), ds*Tai*-1, ds*Tai*-1+10 ng/ml 20E (JH), ds*Tai*-2 or +10 ng/ml 20E (JH) for an additional day.

For each treatment, 15 repeats were set. Three replicates were used to observe the pupation and adult emergence and three replicates were dissected to see the internal organs by allowing the larvae to feed on treated foliage for 3 days (replaced with freshly treated ones each day), and on untreated foliage until reaching the wandering stage. Integument and tracheae were observed and photographed under a light microscope. Dissection and observation were performed from day 4 (continuously feeding dsRNA for 3 days) to day 15 after ecdysis to fourth-instar larvae at an interval of 2 days. For each treatment, 3–4 larvae were dissected at each time point. For extraction of 20E, JH and total RNA for gene expression analysis, a total of nine replicates were collected after continuously fed on treated foliage for 3 days.

### Real-Time Quantitative PCR (qRT-PCR)

For temporal expression analysis, the cDNA templates were derived from the first, second, and third larval instars at an interval of 1 day, and from fourth larval instars at an interval of 8 or 12 h (IxD0/IxH0 indicated newly ecdysed larvae). For tissue expression analysis, RNA templates were derived from the brain-corpora cardiaca-corpora allata complex, ventral ganglion, muscle, trachea, foregut, midgut, ileum, rectum, Malpighian tubules, hemocytes, fat body, and epidermis of the day 4 fourth-instar larvae. For analysis of the effects of treatments, total RNA was extracted from treated larvae. Each sample contained 5–10 individuals and repeated three times. The RNA was extracted using SV Total RNA Isolation System Kit (Promega). Purified RNA was subjected to DNase I to remove any residual DNA according to the manufacturer’s instructions. Quantitative mRNA measurements were performed by qRT-PCR in technical triplicate, using 4 internal control genes (*LdRP4, LdRP18, LdARF1*, and *LdARF4*, the primers listed in Supplementary Table S1) according to our published results (Shi et al., 2013). An RT negative control (without reverse transcriptase) and a non-template negative control were included for each primer set to confirm the absence of genomic DNA and to check for primer-dimer or contamination in the reactions, respectively.

According to a previously described method (Bustin et al., 2009), the generation of specific PCR products was confirmed by gel electrophoresis. The primer pair for each gene was tested with a 10-fold logarithmic dilution of a cDNA mixture to generate a linear standard curve [crossing point (CP) plotted vs. log of template concentration], which was used to calculate the primer pair efficiency. All primer pairs amplified a single PCR product with the expected sizes, showed a slope less than -3.0, and exhibited efficiency values ranging from 2.4 to 2.7. Data were analyzed by the 2^-ΔΔCT^ method, using the geometric mean of the four internal control genes for normalization. Relative transcripts are the ratios of relative copy numbers in treated individuals to control (CK), which are set as 1.

### Quantitative Determination of JH and 20E

Hemolymph was collected from the treated larvae. JH was extracted following the methods described previously (Zhou et al., 2013). Its content (ng per ml hemolymph) was quantified by liquid chromatography tandem mass spectrometry system (Cornette et al., 2008).

20E was extracted according to a ultrasonic-assisted extraction method (Liu et al., 2014), and its titer (ng per g body weight) was analyzed by a liquid chromatography tandem mass spectrometry-mass spectrometry (LC-MS/MS) system using a protocol the same as described (Zhou et al., 2011).

### Data Analysis

We used SPSS for Windows (Chicago, IL, United States) for statistical analyses. The averages (± SE) were submitted to analysis of variance with the Tukey-Kramer test. Since no significant differences in phenotypes between dsRNAs targeting two different regions of *LdTai* gene (ds*Tai*-1 and ds*Tai*-2) were found, we used the results from ds*Tai*-1 (hereafter referred as ds*Tai*) for detailed analyses.

## Results

### Identification of *LdTai*

**FIGURE 1:**
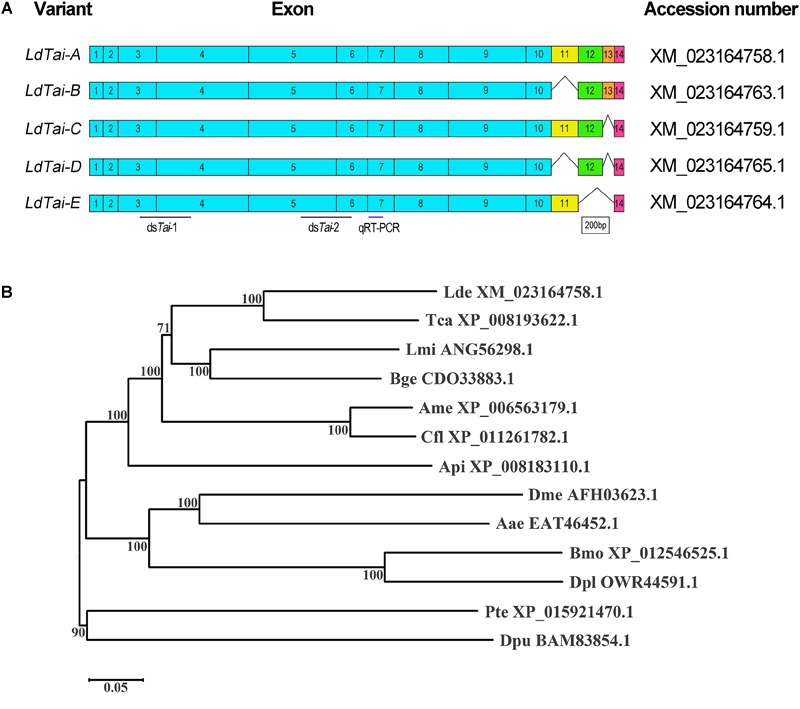
Scheme of the *LdTai* isoforms from *Leptinotarsa decemlineata* **(A)** and phylogenetic analysis of Taiman-like proteins of representative Arthropoda species **(B)**. **(A)** The constitutive exons are shown as boxes. E11, E12 and E13 are alternatively spliced in the isoforms. Black lines point to the region where dsRNA is designed to knock down all *LdTai* isoforms. Blue line indicates the locations used in qRT-PCR. **(B)** The Taiman-like proteins are from two Coleopterans *Leptinotarsa decemlineata* (Lde) and *Tribolium castaneum* (Tca), two Hymenopterans *Apis mellifera* (Ame) and *Camponotus floridanus* (Cfl), two Dipterans *Drosophila melanogaster* (Dme) and *Aedes aegypti* (Aae), two Lepidopterans *Bombyx mori* (Bmo) and *Danaus plexippus* (Dpl), an Orthopteran *Locusta migratoria* (Lmi), a Blattodean *Blattella germanica* (Bge), a Hemipteran *Acyrthosiphon pisum* (Api), an Arachnida *Parasteatoda tepidariorum* (Pte) and a Crustacea *Daphnia pulex* (Dpu). The tree is constructed using the neighbor-joining method based on the full-length protein sequence alignments. Bootstrap analyses of 1000 replications are carried out and bootstrap values > 50% are shown on the tree.

By mining the *L. decemlineata* transcriptome and genome data, five *LdTai* transcripts were found (XM_023164758.1, XM_023164759.1, XM_023164764.1, XM_023164763.1, and XM_023164765.1). *LdTai* gene contains 14 exons. E11, E12, and E13 are alternatively spliced to form the five isoforms. *LdTai-A* (XM_023164758.1) includes all the 14 exons, whereas *LdTai-B* (XM_023164763.1), *LdTai-C* (XM_023164759.1), *LdTai-D* (XM_023164765.1) and *LdTai-E* (XM_023164764.1), respectively, lack E11, E13, E11 and E13, and E12 and E13 (Figure 1A).

Evolutionary relationship of Tai-like proteins derived from 13 species was evaluated. An unrooted phylogenetic tree revealed that Tai-like proteins formed two clades: one from insects, and the other from Arachnida and Crustacea. In insect group, two from Coleopterans, two from Hymenopterans, two from Dipterans and two from Lepidopterans, respectively, clustered together with 100% of bootstrap support. Obviously, *Ld*Tai-A (XM_023164758.1) belonged to Coleopteran cluster (Figure 1B).

Taiman contains four major domains: a bHLH domain, a PAS domain, LxxLL motifs and domain containing several polyglutamine stretches (PolyQ) (Wang et al., 2016). The bHLH, PAS-A, and PAS-B domains are conserved among insect species (Supplementary Figure S1).

### Expression Pattern of *LdTai*

**FIGURE 2:**
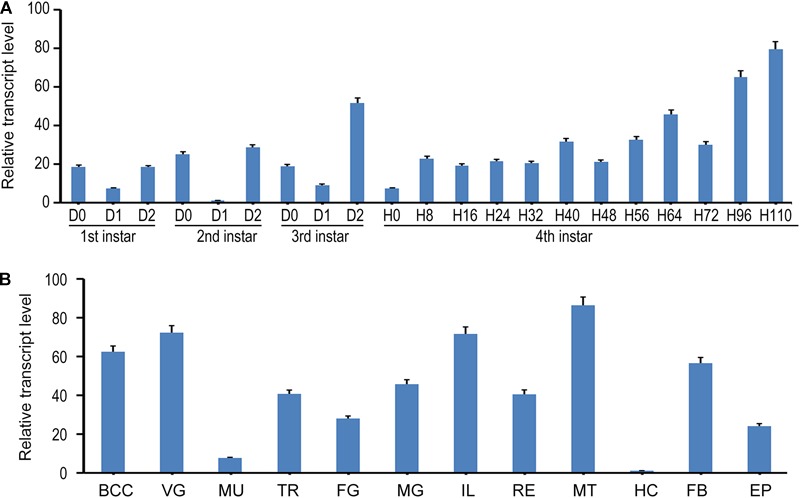
The expression pattern of *LdTai* in *L. decemlineata*. **(A)** Temporal expression analysis. The cDNA templates were derived from the first, second, and third larval instars at the interval of 1 day, and from fourth larval instars at the interval of 8 or 12 h (IxD0/IxH0 indicated newly ecdysed larvae). **(B)** Tissue expression analysis, the cDNA templates are from total RNA extracted from the brain-corpora cardiaca-corpora allata complex (BCC), ventral ganglion (VG), muscle (MU), trachea (TR), foregut (FG), midgut (MG), ileum (IL), rectum (RE), Malpighian tubules (MT), hemolymph (HE), fat body (FB), and epidermis (EP) of the day 4 fourth-instar larvae. For each sample, 3 independent pools of 5–10 individuals are measured in technical triplicate using qRT-PCR. The bars represent 2^-ΔΔCt^ value (± SE) normalized to the geometrical mean of house-keeping gene expression. The relative transcript levels are the ratios of relative copy numbers in different developing stages to larvae at day 1 second-instar stage, or the ratios of relative copy numbers in different tissues to hemocytes (HC).

In the present paper, we designed a pair of primers in E7 to measure the expression of all splicing isoforms (hereafter *LdTai*) by qRT-PCR (Figure 1A). The temporal expression pattern analysis revealed that *LdTai* mRNA was detectable throughout all larval stages. Within the first, second and third larval instars, the expression levels were higher just before and right after the molt than those in the intermediate instar. In the fourth larval instar, the level of *LdTai* gradually rose and a peak occurred 112 h after ecdysis (Figure 2A).

The tissue expression profiles of *LdTai* were also determined. *LdTai* was widely expressed in the brain-corpora cardiaca-corpora allata complex, VG, muscle, trachea, foregut, midgut, ileum, rectum, MT, hemocytes, FB, and epidermis of the day 4 fourth-instar larvae. Its levels were higher in the cardiaca-corpora allata complex, VG, ileum and MT, moderate in the trachea, foregut, midgut, rectum, FB and epidermis, and low in the muscle and hemocytes (Figure 2B).

### Effect of ds*Tai*, Hormone and Their Combination on Larval Performance

**FIGURE 3:**
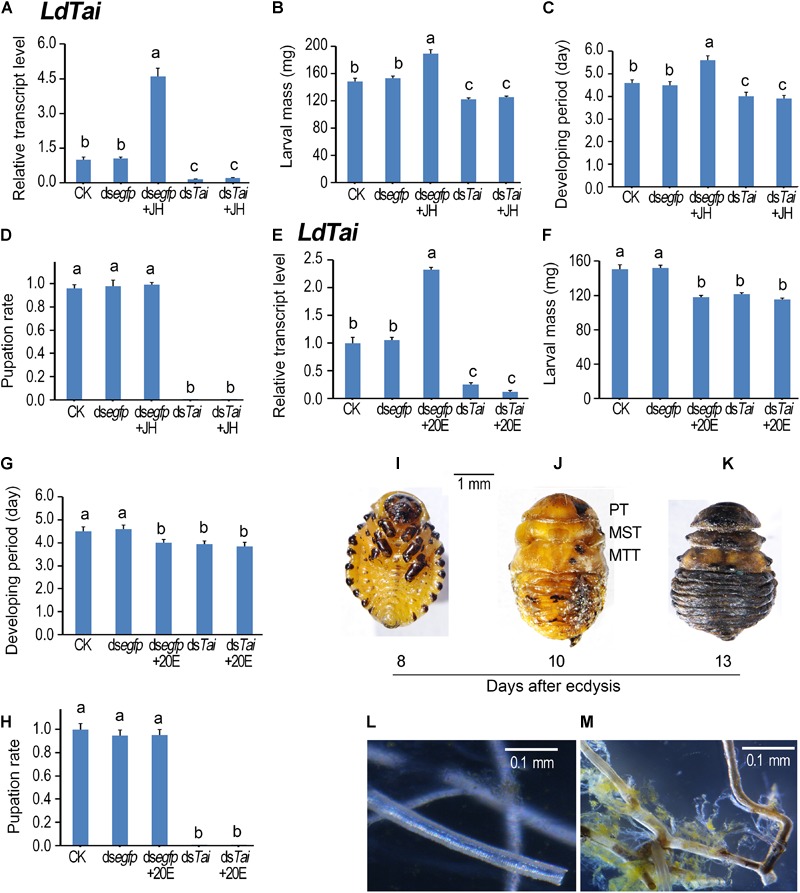
Ingestion of ds*Tai* by the final instar larvae affects larval performance in *Leptinotarsa decemlineata*. The newly ecdysed final instar larvae had ingested PBS (CK)-, ds*egfp*-, JH-, ds*Tai*- and ds*Tai*+JH-dipped leaves, or PBS (CK)-, ds*egfp*-, 20E-, ds*Tai*- and ds*Tai*+20E-immersed foliage for 3 days, and untreated foliage for several days if necessary. The expression levels of *LdTai* **(A,E)** were tested. Relative transcripts are the ratios of relative copy numbers in treated individuals to PBS-fed controls (CK), which is set as 1. The larvae were weighed at an interval of 12 h after treatment to evaluate the maximum weight **(B,F)**. Developing periods were calculated from initiation of bioassay to occurrence of soil-digging behavior **(C,G)**. The pupation rates were recorded during a 4-week trial period **(D,H)**. The bars represent values (± SE). Different letters indicate significant difference at *P*-value < 0.05. The blank controls and ds*egfp*-exposed larvae became prepupae, pupae and adults 5, 7, and 12 days after experiment. Eight days after initiation of the experiment, these *LdTai* RNAi beetle were still wrapped in the old larval cuticles **(I)**. After removal of the apolysed larval cuticle, the *LdTai* RNAi prepupae possess pupal prothorax (PT), mesothorax (MST) and metathorax (MTT) **(J)**. Thirteen days after initiation of the experiment, the *LdTai* depleted prepupae are somewhat withered, dried and darkened **(K)**. The normal tracheae were formed after PBS- and ds*egfp*-fed beetles emerged to adults **(L)**. In contrast, the old tracheal linings still remained in tracheal trunks, some tracheae became brown in *LdTai* knockdown larvae **(M)**.

To dissect the physiological roles of *Ld*Tai, we dietarily introduced each of the two dsRNAs targeting different regions (Figure 1A) of the *LdTai* mRNA (ds*Tai*-1 and ds*Tai*-2) into newly molted fourth-instar larvae, and observed the impacts on larval performance. As no significant differences between ds*Tai*-1 and ds*Tai*-2 were found, we used the results from ds*Tai*-1 (hereafter referred as ds*Tai*) for detailed analyses (Figure 3).

After the fourth larval instars continuously exposed to ds*Tai*-immersed foliage for 3 days, *LdTai* mRNA abundance in the treated larvae significantly decreased, comparing with PBS- and ds*egfp*-exposed larvae (Figure 3A,E). As a result, the average weights of the fully grown larvae were reduced (Figure 3B,F), and the developing periods of the larvae (from the initiation of the bioassay to the occurrence of soil-digging behavior) were shortened (Figure 3C,G). Moreover, all the ds*Tai*-exposed larvae did not normally pupate (Figure 3D,H, 4D). While the PBS- and ds*egfp*-exposed larvae became prepupae, pupae and adults 5, 8, and 13 days after initiation of bioassay, all *LdTai* RNAi larvae showed arrested development (Figure 3I). Eight days after initiation of the experiment, these *LdTai* RNAi beetles were still wrapped in the old larval cuticles (Figure 3I). After removal of the apolysed larval cuticle, the *LdTai* RNAi prepupae possessed pupal prothorax, mesothorax and metathorax (Figure 3J). Thirteen days after initiation of the experiment, the *LdTai* depleted prepupae were somewhat withered, dried and darkened (Figure 3K), and finally died. After PBS- and ds*egfp*-fed beetles emerged to adults, the normal tracheae were formed (Figure 3L). In *LdTai* knockdown larvae, the old tracheal linings still remained in tracheal trunks, some tracheae became brown (Figure 3M).

**FIGURE 4:**
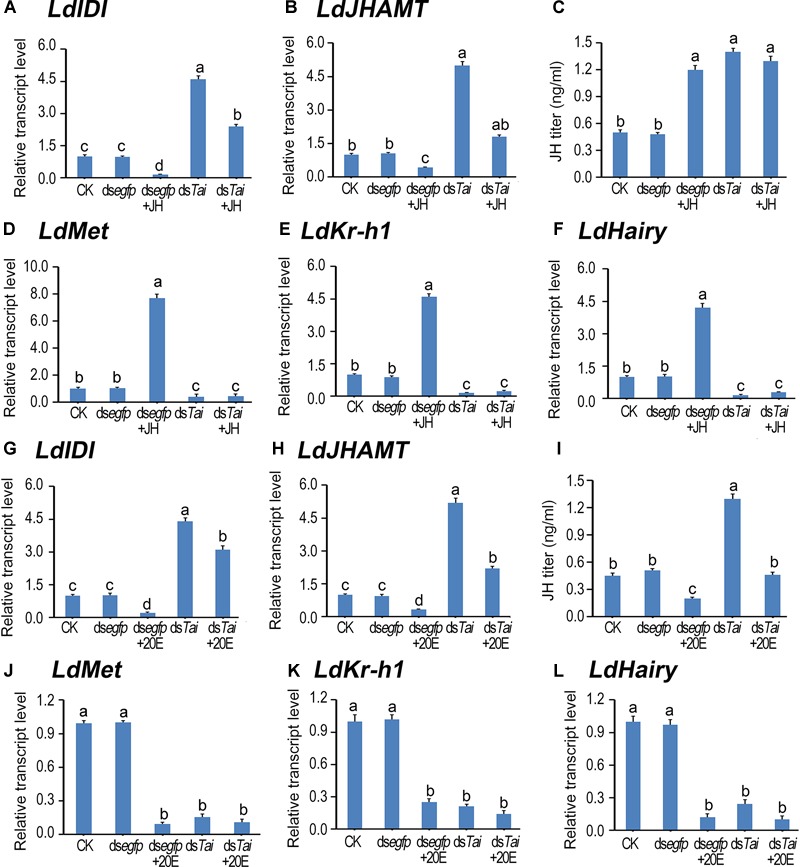
Deletion of *LdTai* disturbs JH signal in *Leptinotarsa decemlineata*. The newly ecdysed final instar larvae had ingested PBS (CK)-, ds*egfp*-, JH-, ds*Tai*- and ds*Tai*+JH-dipped leaves, or PBS (CK)-, ds*egfp*-, 20E-, ds*Tai*- and ds*Tai*+20E-immersed foliage for 3 days. The expression levels of two JH biosynthesis genes *LdIDI* **(A,G)** and *LdJHAMT* **(B,H)**, three JH signaling genes *LdMet* **(D,J)**, *LdKr-h1* **(E,K)** and *LdHairy* **(F,L)** were measured using qRT-PCR. JH titer was tested **(C,I)**. The bars represent average values (± SE). Different letters indicate significant difference at *P*-value < 0.05.

Ingestion of JH or 20E by the PBS-fed fourth-instar larvae activated the expression of *LdTai* (Figure 3A,E). JH ingestion increased the average weight of the fully grown larvae and lengthened the developing period (Figure 3B,C), whereas 20E feeding reduced the average weight of the fully grown larvae and shortened the developing period of the PBS-exposed larvae (Figure 3F,G). Moreover, either JH or 20E did not affect the pupation rate of the PBS-fed larvae (Figure 3D,H, 4D). Ingestion of either JH or 20E by the *LdTai* RNAi larvae did not rescue the decreased expression level of *LdTai* (Figure 3A, 4E), the lowered average weight of the fully grown larvae (Figure 3B,F), the shortened developing period (Figure 3C,G), and the reduced pupation rate (Figure 3D,H).

### Knockdown of *LdTai* Disturbs Hormonal Signals, Non-rescuable by JH or 20E

*LdIDI* and *LdJHAMT* are two biosynthesis genes that encode isopentenyl-diphosphate isomerase and JH acid methyltransferase (Fu et al., 2016; Li et al., 2016), and *LdKr-h1* (*Krüppel homolog 1*) and *LdHairy* are two JH signaling genes (Meng et al., 2018). Knockdown of *LdTai* enhanced JH biosynthesis but reduced JH signaling. Ingestion of JH by PBS-fed larvae decreased the expression levels of *LdIDI* and *LdJHAMT*, raised the JH titer and intensified the expression of *LdKr-h1* and *LdHairy*. In contrast, feeding of 20E by PBS-exposed larvae decreased both JH biosynthesis and JH signaling. However, the reduction of JH signal in the *LdTai* RNAi larvae was non-rescuable by either JH or 20E (Figure 4).

**FIGURE 5:**
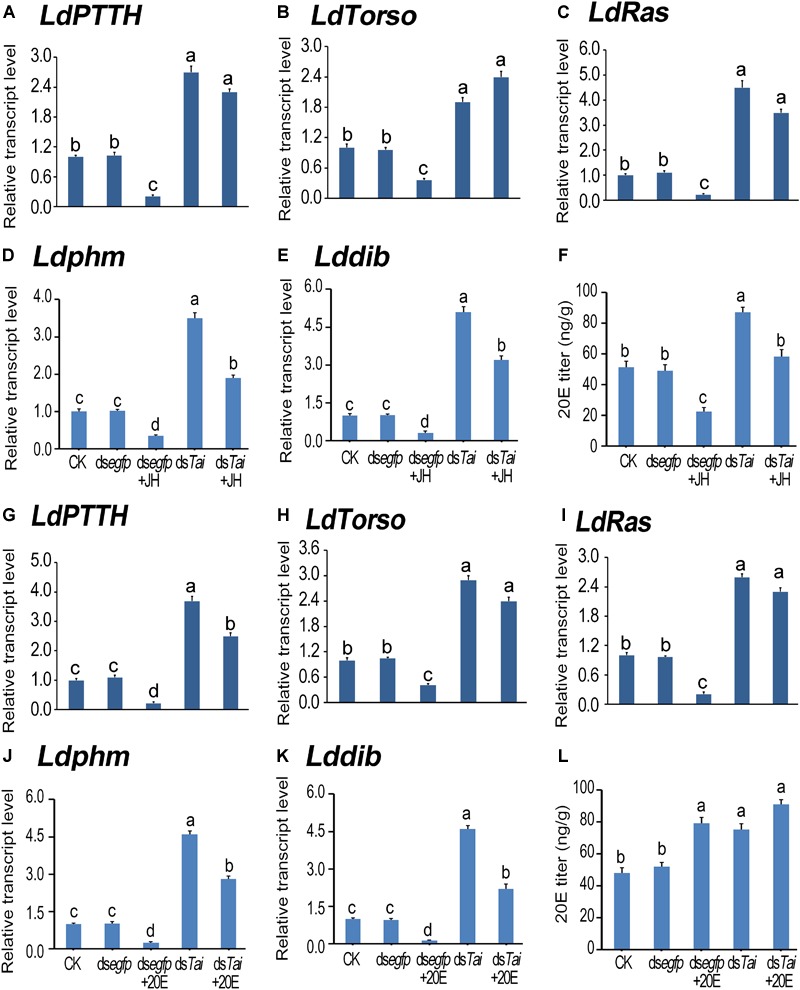
Silence of *LdTai* affects ecdysteroidogenesis in *Leptinotarsa decemlineata*. The newly ecdysed final instar larvae had ingested PBS (CK)-, ds*egfp*-, JH-, ds*Tai*- and ds*Tai*+JH-dipped leaves, or PBS (CK)-, ds*egfp*-, 20E-, ds*Tai*- and ds*Tai*+20E-immersed foliage for 3 days. The mRNA levels of *LdPTTH* **(A,G)**, *LdTorso* **(B,H)**, *LdRas* **(C,I)**, two Halloween genes *Ldphm* **(D,J)** and *Lddib* **(E,K)** were tested using qRT-PCR. 20E titer was determined **(F,L)**. The bars represent average values (± SE). Different letters indicate significant difference at *P*-value < 0.05.

According to the expression peaks (Zhu et al., 2015; Shi et al., 2017), the expression levels of PTTH gene (*LdPTTH*), receptor tyrosine kinase gene *Torso* (*LdTorso*) and a MAPK gene (*LdRas*) were measured after the animals have ingested dsRNA for 3 days. The transcript levels of the three genes were significantly raised in the specimens having ingested ds*Tai* or ds*Tai*+JH (Figure 5A–C), or ds*Tai* or ds*Tai*+20E (Figure 5G–I), whereas their mRNA levels were significantly reduced in the larvae having fed on JH but upregulated in the larvae having fed on 20E, compared with those in the control specimens (Figure 5A–C,G–I).

Influence on PTTH signaling in treated larvae may change ecdysteroidogenesis. Therefore, we tested the expression levels of two ecdysteroidogenesis genes *Ldphm* and *Lddib* (Wan et al., 2013; Kong et al., 2014) and measured 20E titers in the treated larvae. As expected, ecdysteroidogenesis was intensified in the samples having ingested ds*Tai* or ds*Tai*+JH (Figure 5D–F), or ds*Tai* or ds*Tai*+20E (Figure 5J–L).

**FIGURE 6:**
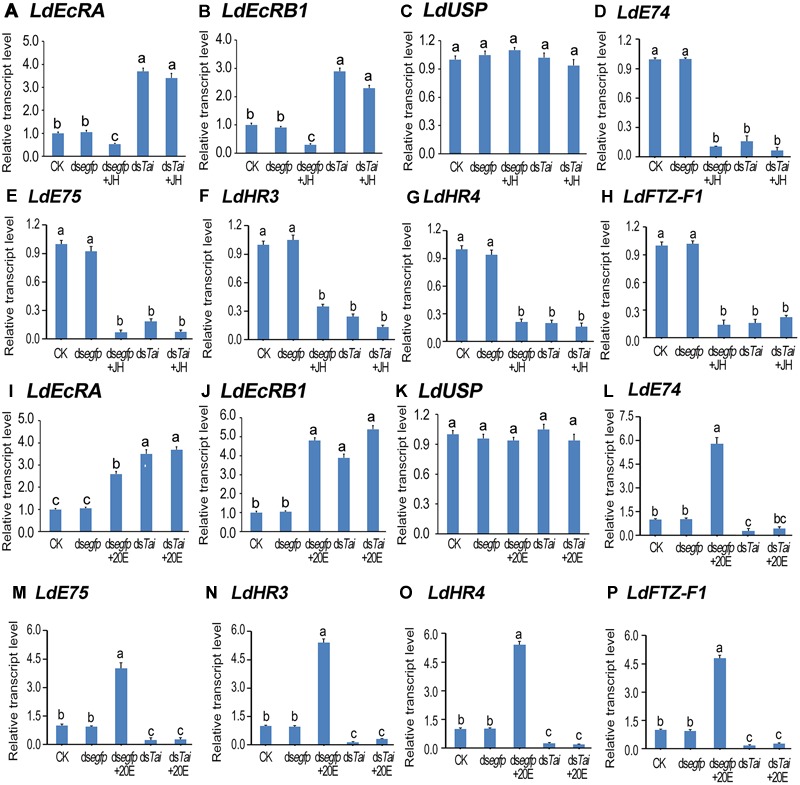
Knockdown of *LdTai* disrupts 20E signaling in *Leptinotarsa decemlineata*. The newly ecdysed final instar larvae had ingested PBS (CK)-, ds*egfp*-, JH-, ds*Tai*- and ds*Tai*+JH-dipped leaves, or PBS (CK)-, ds*egfp*-, 20E-, ds*Tai*- and ds*Tai*+20E-immersed foliage for 3 days. The mRNA levels of eight 20E signaling transcripts *LdEcRA* **(A,I)**, *LdEcRB1* **(B,J)**, *LdUSP* **(C,K)**, *LdE74* **(D,L)**, *LdE75* **(E,M)**, *LdHR3* **(F,N)**, *LdHR4* **(G,O)** and *LdFTZ-F1*) **(H,P)** were tested using qRT-PCR. The bars represent average values (± SE). Different letters indicate significant difference at *P*-value < 0.05.

Subsequently, we determined the expression levels of 20E signaling transcripts *LdEcRA* (Figure 6A,I), *LdEcRB1* (Figure 6B,J), *LdUSP* (Figure 6C,K), *LdE74* (Figure 6D, 6L), *LdE75* (Figure 6E,M), *LdHR3* (Figure 6F,N), *LdHR4* (Figure 6G,O), and *LdFTZ-F1* (Figure 6H,P; Liu et al., 2014; Guo et al., 2015, 2016; Xu et al., 2018a,b, 2019a,b). The levels of *LdEcRA* and *LdEcRB1* were elevated; the mRNA level *LdUSP* of remained unchanged; whereas the expression levels of *LdE74, LdE75, LdHR3, LdHR4*, and *LdFTZ-F1* were lowered. Therefore, silencing *LdTai* attenuated 20E signaling.

Ingestion of JH by control larvae diminished both ecdysteroidogenesis and 20E signaling. Feeding of 20E by control larvae heightened 20E titer and reinforced 20E signal. However, neither JH nor 20E restored the decreased 20E signal in the *LdTai* RNAi larvae (Figure 5, 6).

## Discussion

Based on findings obtained from RNAi and hormone rescuing experiments, we established in the present paper that Tai was critical for the regulation of metamorphosis by mediating both JH and 20E signals in *L. decemlineata*.

### *Ld*Tai Mediates JH Signaling

In *L. decemlineata*, the expression levels of *LdTai* were higher right after the molt than those in the intermediate instar within the first, second and third larval instars (Figure 2A). Comparably, within each instar the JH titers appear to rise shortly before or right after the molt, and then drop sharply in *L. decemlineata* (Meng et al., 2015) and the drywood termite *Cryptotermes secundus* (Korb et al., 2012). It appears that the mRNA levels of *LdTai* are positively correlated with circulating JH titers in *L. decemlineata*. Moreover, JH ingestion enhanced the transcription of *LdTai* in *L. decemlineata* (Figure 3A), as well as in *B. germanica* (Lozano et al., 2014) and *B. dorsalis* (Liu et al., 2017). Upregulation of *Tai* transcription in response to JH in aforementioned insect species indicates that Tai is the functional receptor of JH.

Our findings in this study revealed that knockdown of *Tai* inhibited larval growth and shortened larval developing period in *L. decemlineata* (Figure 3B,C,F,G). Similar phenotypes have been documented in *T. castaneum Tai* RNAi larvae (Bitra et al., 2009). Moreover, we found that dietary supplement with JH failed to rescue the lowered average weight and the shortened developing period in *LdTai* RNAi larvae, even though JH ingestion increased the average weight and lengthened the developing period in control *L. decemlineata* larvae. In agreement with these results, our previous results disclose that knockdown of *LdMet* reduces body size, the reduction cannot be restored by JH in *LdMet* RNAi larvae (Meng et al., 2018). The similar regulation of Met (Meng et al., 2018) and Tai (this study) on larval growth indicates that the two bHLH-PAS transcription factors form a heterodimer to mediate JH signaling.

We directly analyzed the influences of *LdTai* silencing on JH signaling in the present paper, and found that silencing *LdTai* decreased the expression of *Met*. It is suggested that Tai may mediate JH to trigger its receptor expression. Moreover, RNAi *LdTai* repressed JH signal. The repression was non-rescuable by JH in the *LdTai* RNAi larvae (Figure 4). Similarly, RNAi of *Tai* significantly reduced the mRNA levels of *Met, Kr-h1* and Broad complex (*BrC*) in *B. germanica* (Lozano et al., 2014), and *Kr-h1* in *P. apterus* (Smykal et al., 2014a). This result again supports that Tai mediates JH signaling.

Our previous results show that knockdown of *LdMet* enhances the transcription of *LdPTTH*. As a result, ecdysteroidogenesis is activated (Meng et al., 2018, 2019). In the present paper, we found that silence of *LdTai* also raised the expression levels of three PTTH signaling genes. As a result, ecdysteroidogenesis was intensified in *LdTai* RNAi larvae (Figure 5, 6).

All these findings in this study support that Tai mediates JH signaling in *L. decemlineata*.

### *Ld*Tai Transduces 20E Signaling

The high *LdTai* levels just before the molt within young larval instars and the peak at 112 h after third-fourth instar ecdysis (Figure 2A) are positively correlated with circulating 20E titers in *L. decemlineata* (Smagghe et al., 1995). Moreover, ingestion of 20E stimulated the transcription of *LdTai* (Figure 3E). It can accordingly be postulated that Tai is the transducer of 20E.

In this study, we found that all *LdTai* RNAi larvae were wrapped in the old larval exuviae in *L. decemlineata*, with abnormal pupation and uncompleted trachea ecdysis (Figure 3H–M). Likewise, some *Tai* mutants in *D. melanogaster* are lethal at larval stages (Perrimon et al., 1996; König et al., 2011; Wang et al., 2016). In *T. castaneum*, metamorphosis fails and all beetles eventually die when Tai is depleted at penultimate and final instar larval stages (Bitra et al., 2009). In hemimetabolan insects *B. germanica* (Lozano et al., 2014) and *P. apterus* (Smykal et al., 2014a), removal of Tai disrupts nymphal ecdysis, and causes 100% mortality. The pharate animals of *P. apterus* undergo apolysis but are unable to shed the old cuticle (Smykal et al., 2014a). Accordingly, disrupted ecdysis in *Tai* RNAi larvae reflects that Tai may transduce 20E message in *L. decemlineata* (this study), as well as other holometabolous and hemimetabolous insect species (Perrimon et al., 1996; Bitra et al., 2009; König et al., 2011; Lozano et al., 2014; Smykal et al., 2014a; Wang et al., 2016).

Moreover, we unveiled that silencing *LdTai* intensified ecdysteroidogenesis (Figure 5) and the expression of *EcR*, but declined 20E signaling. The decline could not restore by 20E ingestion in *LdTai* RNAi larvae (Figure 6). Since nuclear receptor coactivators are essential for steroid-dependent transactivation of genes in addition to the availability of the hormone and the expression of its receptor (Tetel, 2009), our results strongly support that Tai acts as a coactivator of ecdysone receptor components during larval-pupal metamorphosis of *L. decemlineata*. Similarly, RNAi of *BgTai* significantly reduced the mRNA levels of *EcR, USP* and *E75A* in *B. germanica* (Lozano et al., 2014). In *A. aegypti*, knockdown of *Tai* reduces the transcript levels of four 20E responsible genes *E75A, E74B, vitellogenin, vitellogenic carboxypeptidase* in the FB of adult females (Zhu et al., 2006; Liu et al., 2018). Thus, our data afford an indication that Tai mediates 20E pathway.

It is well known that a high level of 20E in Holometabolan insects such as *M. sexta, D. melanogaster, Bombyx mori*, and *T. castaneum* stimulates the production but prevents the release of ecdysis triggering hormone (ETH). On the other hand, the high 20E level sensitizes the central nervous system to ETH by expressing the ETH receptor (ETHR) (Adams and Žitňan, 1997; Žitňan et al., 2002, 2003, 2007; Kim et al., 2006; Arakane et al., 2008; Bai et al., 2011). However, acquisition of Inka cell competence to release ETH requires a decline of 20E level and timely *FTZ-F1* expression a few hours prior to ecdysis (Zitnan et al., 2007). In the present paper, however, 20E titers were higher and expression levels of *FTZ-F1* were lower in *LdTai* RNAi larvae, this may inhibit ETH release and impair pupation.

### Tai May Be Involved in Other Signaling Pathways

In *A. aegypti*, using a yeast two-hybrid system, it has been found that Tai interacts with the transcription factor FTZ-F1. Therefore, *Aa*Tai may serve as a coactivator of FTZ-F1, and is named as FTZ-F1-interacting steroid receptor coactivator (FISC) (Zhu et al., 2006). Moreover, Tai promotes *Drosophila* adult intestinal stem cell proliferation as a coactivator of Yorkie in the Hippo pathway (Ren et al., 2010; Shaw et al., 2010; Wang et al., 2016). In *B. germanica*, knockdown of some *Tai* isoforms significantly reduces the expression of an insulin gene *BgILP-1* (Lozano et al., 2014), suggesting that Tai may be involved in the insulin signaling pathway. Our results in the present paper do not exclude that Tai may act as a player in other signaling pathways.

## Conclusion

Our results indicate that Tai mediates both JH and 20E signaling pathways during larval-pupal-adult transition in *L. decemlineata*, perhaps acting as transcription factor and transcription cofactor, respectively. Therefore, our results uncover a link between JH and 20E pathways in metamorphosis. Compatibly, a bHLH-PAS family protein aryl hydrocarbon receptor nuclear translocator protein (ARNT) can also function as either transcription factors or transcription cofactors, depending on the circumstances. Upon binding of environmental pollutants, a bHLH-PAS domain protein aryl hydrocarbon receptor (AHR) translocates from the cytoplasm to the nucleus to form the ARNT-AHR heterodimer. The dimer binds directly to the xenobiotic response element and activates expression of proteins involved in xenobiotic metabolism (Swanson et al., 1995). ARNT can also serve as a coactivator of estrogen receptor (ER)-dependent transcription, by recruitment to ER target gene promoters and physical interaction with ER (Brunnberg et al., 2003).

## Data Availability

The raw data supporting the conclusions of this manuscript will be made available by the authors, without undue reservation, to any qualified researcher.

## Ethics Statement

The research project was conducted on invertebrate species that are not subjected to any specific ethical issue and legislation.

## Author Contributions

Q-YX and G-QL designed the research. Q-YX, J-LD, and L-LM performed all of the experiments. W-CG provided the materials. Q-YX, J-LD, and G-QL analyzed the data. W-CG and G-QL wrote the manuscript.

## Conflict of Interest Statement

The authors declare that the research was conducted in the absence of any commercial or financial relationships that could be construed as a potential conflict of interest.
