# Supplementary material for: Importance of Taiman in Larval-Pupal Transition in Leptinotarsa decemlineata
Source: Front Physiol. 2019 Jun 13;10:724. doi: 10.3389/fphys.2019.00724 (PMC6584964; doi:10.3389/fphys.2019.00724)
Supplement: Supplementary file 1 [file Table_1.DOC]

Supplementary data

**Importance of Taiman in larval-pupal transition in *Leptinotarsa decemlineata***

Qing-Yu Xu^1^, Pan Deng^1^, Li-Li Mu^1^, Kai-Yun Fu^2^, Wen-Chao Guo^3^, Guo-Qing Li ^1^ *

1. Education Ministry Key Laboratory of Integrated Management of Crop Diseases and Pests, College of Plant Protection, Nanjing Agricultural University, Nanjing 210095, China
2. Institute of Plant Protection, Xinjiang Academy of Agricultural Sciences, Urumqi 830091, China; Key Laboratory of Intergraded Management of Harmful Crop Vermin of China North-western Oasis, Ministry of Agriculture, China
3. Institute of Microbiological Application, Xinjiang Academy of Agricultural Sciences; Urumqi 830091, China

**Table S1. Primers used in dsRNA synthesis and qRT-PCR**

| **Fragment name** | **Forward primer** | **Reverse primer** |
| --- | --- | --- |
| **dsRNA synthesis** |  |  |
| ds*Tai1* | GAAAGTGCCTCCCAAGCG | CTCGTAGCGGCTGACCC |
| ds*Tai2* | CCAGGTTCTCCTTTAGCCC | GCCTTCTTGTCTTCCTCGCT |
| ds*egfp* | AAGTTCAGCGTGTCCG | CACCTTGATGCCGTTC |
| **qRT-PCR** |  |  |
| q*LdTai* | CCTCCGGGAACGTAATAAGA | CTTTCAGGTATTGGCGGAAT |
| q*LdIDI* | AAGCCAGCAAGAAAGGATGT | CCGGGTAGGTAATCTTCTGG |
| q*LdJHAMT* | GGAAGTGGAGATGGCAAGTT | CTACCAACGAGTTTCCCGAT |
| q*LdMet* | GCGACTCCGCTAACAGTGTA | AGATCCCTTCCTGGTTGTTG |
| q*LdKr-h1* | CGATGGTCTCAGAAGAAGGG | AATTGGGAGGCGTAACAGTC |
| q*LdHairy* | CGGAACCAATGGACACTATG | ACACATTTCCTCACGACCAA |
| q*LdPTTH* | TCCAAACTGGATCCTGTCAA | CACAGAGGAGGTTTCGTCAA |
| q*LdTorso* | CCAAAGTGCAGACTCCTCAA | CTTTGTTGCTCCCTCTTTCC |
| q*LdRas* | GACGTACAGGGAGCAGATCA | CTTGGCGACCTCTCTAGCTT |
| q*Ldphm* | GGAGAAAACTCGGAAAAGTGAAGA | CACCAAATAAATCAGCCAGCAA |
| q*Lddib* | ATCCTCAGAGAAGAAGCCATTTAGC | CGAAACAATCCACTCCCGAATAACC |
| q*LdEcRA* | CGGGTTCAATAACAGTGTCG | ATAAGGTTGCGAAGGTGGTC |
| q*LdEcRB1* | GGGAGTGCTAGTGTTGTGGA | GGATTAGACGCTCCCTACGA |
| q*LdUSP* | TCGTCCTTTGCCGTTCTTCTTG | CGACAAAGAAACAGATGCCAGTAC |
| q*LdE74* | ATGCCATTTATCGACAACGA | ACCTCCTGAAGTACCGGTTG |
| q*LdE75* | CCAACTCCAGATGCAGCTTA | AATGATTTCGCCAACATTGA |
| q*LdHR3* | GCATCATCCAGCAAATCATC | CTGATCGTCTTGCGACAACT |
| q*LdHR4* | ATTATGACCCTTACGCGGACC | GCGACAGATTCGCCATCAC |
| q*LdFTZ-F1* | GGCTAATCAGGCCTCCAG | CATGGTTTGCTGGCAACTAC |
| *qLdRP18* | TAGAATCCTCAAAGCAGGTGGCGA | AGCTGGACCAAAGTGTTTCACTGC |
| *qLdRP4* | AAAGAAACGAGCATTGCCCTTCCG | TTGTCGCTGACACTGTAGGGTTGA |
| *qLdARF1* | CGGTGCTGGTAAAACGACAA | TGACCTCCCAAATCCCAAAC |
| *qLdARF4* | GTGCTCGTGAACCATGTGAA | AACCTCCAATCCCTCGTGAA |


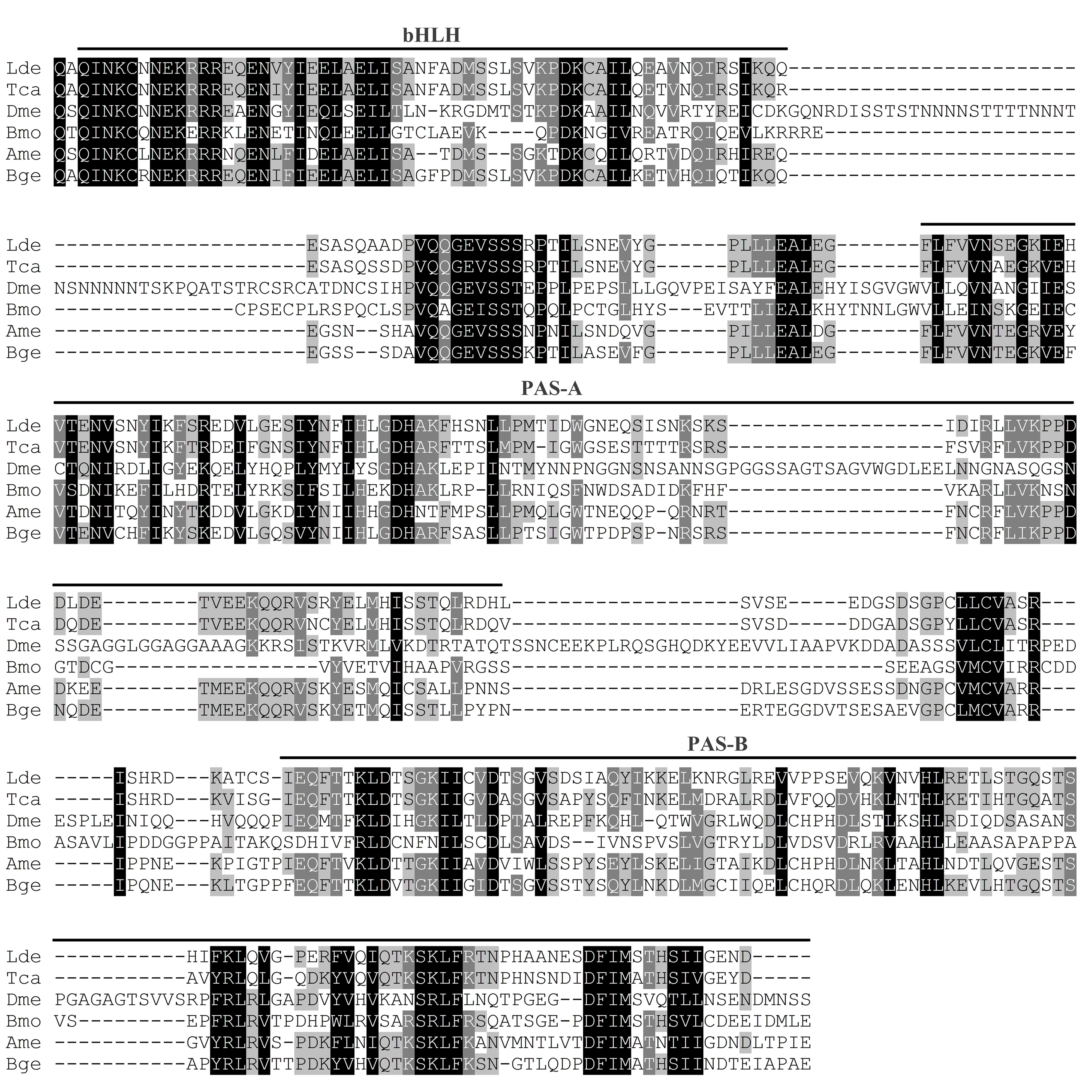


Figure S1. Alignment of bHLH and PAS regions of Taimans from six insects. Amino acid sequences of Taiman proteins derive from *Leptinotarsa decemlineata* (Lde), *Tribolium* *castaneum* (Tca, XP_008193622.1), *Drosophila* *melanogaster* (Dme, AFH03623.1), *Bombyx* *mori* (Bmo, XP_012546525.1), *Apis* *mellifera* (Ame, XP_006563179.1) and *Blattella* *germanica* (Bge, CDO33883.1). Increasing background intensity (from light to dark) indicates an increase in sequence similarity. Gaps have been introduced to permit alignment. The bHLH, PAS-A and PAS-B domains are highlighted by black lines.
